# Between Regional Recommendations and National Implementation: An Analysis of the East African Community Partner States’ Legislative Responses to TRIPS Obligations

**DOI:** 10.1007/s40319-023-01318-7

**Published:** 2023-05-03

**Authors:** Olugbenga A. Olatunji

**Affiliations:** grid.1013.3 0000 0004 1936 834X PhD; Lecturer, The University of Sydney Law School, Camperdown Campus, Sydney, Australia

**Keywords:** TRIPS Agreement, Access to medicines, East African Community, EAC and TRIPS flexibilities, Patent laws (Kenya, Uganda, Tanzania, Rwanda, Burundi)

## Abstract

With the access-to-medicines conundrum facing its populations, the East African Community has adopted a policy framework which promotes a collective approach to resolving the access gap in the region. To this end, crucial policy documents on the implementation of TRIPS obligations, harmonisation of drug regulation and boosting regional manufacturing capacity have been adopted. This paper is a case study of the regional policy on the implementation of TRIPS obligations, specifically examining the extent to which partner states’ implementation of TRIPS obligations mirrors the regional recommendations. The paper finds that, while many partner states follow the regional recommendations on implementing TRIPS obligations, coherence remains a big challenge. This, the paper concludes, may affect the overall usefulness of a regional approach to solving the access conundrum.

## Introduction

The COVID-19 pandemic has laid bare the inefficiency of the existing arrangement for meeting access-to-medicines needs in low- and middle-income countries (LMICs) – substantially relying on goodwill donations from high-income countries.^1^ This problem predates the pandemic, which explains why the access-to-medicines policy landscape is already dotted with numerous national and regional policies aimed at finding lasting solutions.^2^ The East African Community (EAC) offers a perfect example in this regard, having adopted access-to-medicines-focused policies such as the EAC policy on TRIPS flexibilities (and an accompanying Protocol),^3^ a pharmaceutical manufacturing plan,^4^ a policy on medicines registration,^5^ and a policy on anti-counterfeiting.^6^ Collectively, these policies offer model recommendations for legislative adoption among partner states, the expected result being an enhanced regional pharmaceutical production capacity for improving access to medicines in the region.

The EAC has seven partner states overall, five of which (Kenya, Uganda, Rwanda, Tanzania, Burundi) are long-time members, while two (South Sudan – September 2016 and Democratic Republic of Congo – July 2022) have recently joined. Similarly, all partner states (except South Sudan)^7^ are signatories to the World Trade Organization (WTO)’s Agreement on Trade-Related Aspect of Intellectual Property Rights (TRIPS)^8^ and are therefore required to incorporate TRIPS minimum standards into their laws. The EAC policy on TRIPS flexibilities provides partner states with implementation options aimed at maximising the use of TRIPS flexibilities for improved access. Overall, the EAC policy on TRIPS flexibilities aggregates ten flexible TRIPS obligations which partner states could construe through an access-to-medicines lens (obligations which were subsequently enacted into a draft EAC Protocol on TRIPS Flexibilities).^9^ These obligations are a transition period, patentability criteria, exclusion from patentability, research exception, *Bolar* exception, test data protection, disclosure requirement, opposition procedure, parallel importation, and compulsory licence. Cumulatively, these recommendations are aimed at expanding available policy spaces within the region with the expected long-term benefit being the enhancement of regional pharmaceutical production capacity. The gravamina of these recommendations have been extensively critiqued elsewhere.^10^

This paper aims at examining the extent to which actual implementation in leading EAC partner states (the old five – Kenya, Rwanda, Uganda, Burundi and Tanzania) reflects the regional recommendations expressed in the policy and the Protocol. This exercise is particularly germane since a harmonised approach to implementation is a *sine* *qua* *non* for the success of any regional approach. As already mentioned, both South Sudan and the Democratic Republic of Congo have only recently joined the EAC; hence, their exclusion. To achieve its aim, this paper employs both doctrinal and interviewing research methods – the former in analysing relevant primary and secondary source materials; the latter in bridging the gap between theoretical policy statements and actual practices. The interview data used were part of a bigger pool of interview data collected by the author in 2018 as part of his PhD fieldwork.^11^ Stakeholders engaged in semi-structured interviews included representatives of pharmaceutical firms, patent offices, procurement agencies, and drug regulation bodies.^12^

It should be noted that while the analysis in this paper relies on the intellectual property laws of five partner states, interviews were only conducted in four (Kenya, Rwanda, Tanzania, and Uganda) – Burundi was left out because it was engulfed in political upheaval at the time of interview. Similarly noteworthy is that, even though five partner states are considered, six IP laws are evaluated. This is occasioned by the peculiar constitutional arrangement in Tanzania, where IP law is not a Union (i.e. federal) matter, thereby resulting in both Tanzania-Mainland and Tanzania-Zanzibar having separate IP laws.^13^

Following from the foregoing background, Section 2 of this paper compares national legislative responses among partner states with regional recommendations, using the ten flexible obligations drawn from the EAC policy/protocol on TRIPS flexibilities as sub-sections. Each subsection features a recap of the regional recommendations on the TRIPS obligation under review, before proceeding to benchmark those recommendations against partner states’ implementation. Comparative tables are used throughout for the purpose of clarity. Some partner states, contrary to the regional recommendations, incorporate TRIPS-plus obligations into their laws. Section 3 examines this trend, flagging how contradictory it is to the overall objective of improving access to medicines in the region. Conclusion is drawn in Section 4, where the significance of a common approach to the success of the regional effort is underscored.

## Implementation of TRIPS Obligations in EAC Partner States – A Comparative Discussion

The EAC policy/protocol on TRIPS flexibilities principally aims at charting a coherent access-to-medicines-friendly interpretative course for partner states to adapt and adopt while incorporating TRIPS obligations. This section assesses the extent to which partner states reflect regional recommendations in their implementation. Each of the ten flexible obligations under the EAC policy/protocol is analysed for this purpose.

### Transition Period

Transition period means the time allowance available to different categories of WTO members (developed, developing, and least developed) to implement TRIPS obligations domestically.^14^ The dates stipulated for developed and developing countries having passed, only least developed countries (LDCs) could invoke the transition period as a flexibility – in the EAC case, this will be Uganda, Tanzania, Rwanda and Burundi. In implementing this obligation, the EAC Protocol on TRIPS Flexibilities recommends the following (Table 1).

**Table 1:** Transition period (regional recommendations)

S/N	Recommendations	EAC Protocol
1	Exclude patent protection for pharmaceutical products until 2016 & later extensions	s 2(1)
2	Exclude patent protection for pharmaceutical processes until 2016 and later extensions	s 2(1)
3	Abolish mailbox system (if already in existence)	s 2(2)

Reference in the above recommendations to “later extensions” is very crucial as it ensures that, like other WTO LDCs, the IP laws of EAC LDCs are well-positioned to take advantage of any subsequent extensions that may be approved by the Council for TRIPS.^15^ This recommendation would later prove justified when the pharmaceutical exemption, initially slated to end in 2016, was extended by the Council to 1 January 2033.^16^

So, what does the incorporation of the transition period look like among partner states? An overview of the national responses indicates that no partner state completely complies with these recommendations: Starting with Uganda and Zanzibar, patent protection for pharmaceutical products is excluded until 1 January 2016 or any other date as may subsequently be applicable – both in line with EAC recommendations.^17^ Zanzibar also excludes patents for pharmaceutical processes as recommended above, but Ugandan law is silent on this, indicating that process patents may be sought for pharmaceuticals in Uganda.^18^ Both partner states, however, disregard the recommendation that the obligation in respect of mailbox applications be excluded, with the practical implication that mailbox patent applications could be filed in both countries.^19^

The responses of Rwanda and Burundi are similar: both, in partial compliance with the EAC recommendation, exclude patent protection for pharmaceutical products.^20^ The Rwandan provision, however, takes a generalised approach by excluding pharmaceutical products from patent protection without specifically envisaging a possible future extension of the LDC transition period.^21^ In contrast, the Burundian provision disadvantages the use of this flexibility beyond 2016 by expressly specifying 2016 as the end date for excluding pharmaceutical products from patentability – this is in contradiction with the EAC suggestion.^22^ While both partner states depart from the EAC position, the Burundian approach is more likely to have a damaging consequence for access to medicines. For instance, by not providing for any end date, Rwanda is able to benefit from subsequent extensions to the pharmaceutical exemption. Such continued use will only abate if either the Council decides not to extend the exemption, or if Rwanda’s status as an LDC later changes. On the other hand, the Burundian approach implies that patent protection for pharmaceutical products has become available in Burundi since the stipulated date in 2016.

On other recommendations, since process patent protection is not expressly excluded, it is impliedly available for pharmaceuticals in Rwanda and Burundi; in the same way, non-implementation of the mailbox system is a clear indication of the non-availability of the system in both partner states. Tanzania-Mainland, the last partner state, complies with the EAC recommendation on mailbox applications, although it ignores others by making patent protection available for pharmaceutical products and processes.

Partner states’ responses are tabulated below.

Table 2 provides a snapshot of the inconsistent implementation approaches among partner states. One prominent instance deserving further comment is the availability of patent protection for pharmaceutical processes in most EAC LDCs. While this contradicts regional recommendations, it is not entirely antithetical to the regional objective of broadening the public domain and promoting local research in pharmaceutical innovations.^23^ In fact, this approach (i.e. availability of patent protection for pharmaceutical processes) may spur research culture regionally for pharmaceutical inventions by encouraging pharmaceutical firms or researchers in the region to reverse-engineer pharmaceutical products/inventions (for which patent protection is currently not available) in search of new and better manufacturing processes (which currently enjoy patent protection). It is reasonable to expect that, in the long run, the investment of more research time and resources in reverse-engineering patent-ineligible pharmaceutical products in search of patent-eligible manufacturing processes may invariably deepen local researchers’ understanding of the chemical composition of the pharmaceutical inventions themselves. This could eventually, though gradually, boost local researchers’ skillsets and put them on the right trajectory for substantive pharmaceutical innovations. This approach had worked for India – patents were only available for pharmaceutical processes and not products. India grew its generic capacity during this era and before the commencement of the compulsory product patent regime introduced under TRIPS.^24^

**Table 2:** Transition period (national implementation)

Partner states	Pharma product	Pharma process	2016 as original date	Subsequent extensions	Mailbox application
Uganda	Excluded	Available	Included	Included	Available
Zanzibar	Excluded	Excluded	Included	Included	Available
T-Mainland	Available	Available	–	–	–
Rwanda	Excluded	Available	–	–	–
Burundi	Excluded	Available	Included	–	–

Finally, it is pertinent that partner states adopt, as much as possible, a harmonised approach to implementing this flexibility so that technological development can occur across the region at a relatively equal pace. According to TRIPS, a transitional period is conceded to WTO LDC members to assist them overcome “economic, financial and administrative constraints”, and create a “… viable technological base.”^25^ As current evidence shows that technological capacity for pharmaceutical manufacturing in the region is significantly limited to basic generic formulations,^26^ partner states should use the transition period to deny patents for pharmaceutical products and to abolish or exclude a mailbox system for as long as their right to do so under TRIPS subsists. This will provide a window within which partner states’ governments could implement complementary policies needed to consolidate existing manufacturing capacity.

### Patentability Criteria

Under TRIPS, whether an invention is patentable is assessed using *inter* *alia* three omnibus criteria of novelty, inventive step, and industrial application.^27^ Because these criteria are couched in general terms, WTO members have invoked them as policy tools to prosecute different innovation strategies: for instance, countries aiming for increased innovative capacity have construed these requirements strictly so as to create a public domain that is permissive of reverse-engineering.^28^ Conversely, countries with significant innovative capability have interpreted the same requirements more permissively to create room for more patents to be granted.^29^ The EAC has opted for the former approach: an easily predictable choice given the region’s desire to create a public domain where nascent local researchers could catch up on the technology race.

The flexibility that patentability requirements provide could be used by all partner states, notwithstanding the fact that most EAC LDCs do not offer patent protection for pharmaceutical products. This is more so since most EAC LDCs offer protection for pharmaceutical processes; hence, they can construe patentability criteria strictly to exclude processes which are not deserving of protection. Additionally, extending this flexibility to EAC LDCs will serve a futuristic purpose since the transition period which currently exempts patent protection for pharmaceuticals is time-bound and may cease anytime. Table 3 outlines the EAC recommendations on patentability criteria.

**Table 3:** Patentability criteria (regional recommendations)

S/N	Recommendations	EAC Protocol
1	Assess "novelty" against "worldwide prior arts"	s 5(1)
2	Measure "inventive step" through "highly skilled person"	s 5(2)
3	Construe "industrial application" strictly (useful in industry) Grant patent only to "research tools" with "specific use"	s 5(3)

In implementing the patentability criteria, partner states have largely followed the regional recommendations. As such, all partner states construe “novelty” strictly by testing claimed inventions against worldwide prior arts;^30^ provide that patents should only be granted to inventions which are applicable within the industry;^31^ but none of them implement the recommendation that patents should only be made available to research tools with “specific uses”. Furthermore, most partner states – Kenya,^32^ Uganda,^33^ Rwanda,^34^ Tanzania-Mainland^35^ and Burundi^36^ – adopt the standard of an “ordinarily skilled person” (cf. “highly skilled person” recommended by EAC) for assessing “inventive step”. Only Zanzibar applies the EAC recommended standard.^37^ Table 4 tabulates national responses among partner states.

**Table 4:** Patentability criteria (national implementation)

Partner states	Novelty	Inventive step	Industrial application	Specific use for research tools
Kenya	Worldwide	Ordinary skill	Useful in industry	No provision
Uganda	Worldwide	Ordinary skill	Useful in industry	No provision
Zanzibar	Worldwide	High skill	Useful in industry	No provision
T-Mainland	Worldwide	Ordinary skill	Useful in industry	No provision
Rwanda	Worldwide	Ordinary skill	Useful in industry	No provision
Burundi	Worldwide	Ordinary skill	Useful in industry	No provision

One missing implementation from Table 4 is failure by partner states to confine the patentability of research tools to those having specific uses. The nature of research tools (e.g. laboratory equipment, antibodies, chemical reagents, etc.) is such that their patentees only benefit when other researchers research with them.^38^ This peculiar feature makes research tool patents very easy to infringe. One possibly unintended consequence of disregarding the regional recommendation is that patent offices in partner states are now mandated to grant patents to research tools asserting multiple uses with the concomitant result that these patents could be used to hinder further research. This could lead to research tool patentees holding out in granting voluntary licences to researchers who may be interested in using their tools.

It is equally interesting that most partner states prefer to assess “inventive step” using the “ordinarily skilled” standard as opposed to the regionally recommended “highly skilled” standard. Apart from the regional approach failing to provide any guideline on what capability is to be ascribed to this nominal person, assessing “inventive step” using such a standard is unrealistic.^39^ That most partner states favour the “ordinary skill” approach attests to the unpopularity of, and the difficulty in, applying this proposed alternative; it also affirms the criticism levelled against the approach elsewhere.^40^ Kenya, for example, is the only partner state with a *Guidelines* *to* *Patenting* document and, according to these *Guidelines*, a person skilled in the art “should be presumed to be an ordinary practitioner aware of what was common general knowledge in the art at the relevant date … .”^41^ Not only is this approach more realistic but it is also workable and succinct enough to address the dreaded issue of low-quality inventions.

### Exclusion from Patentability

The use of this obligation as a national policy tool derives from TRIPS’s non-mandatory provisions on excludable subject-matter.^42^ WTO members are therefore free, within the limit permissible under TRIPS, to exclude other subject-matter from patentability.^43^ Relying on this, the EAC recommends a two-category approach to implementing this obligation – see Table 5.

**Table 5:** Exclusion from patentability (regional recommendations)

S/N	Recommendations	EAC Protocol
1	Omnibus exclusion provision allowing partner states to exclude subject-matter as they deem fit	s 4
2	Specific exclusions Natural substances, isolated or purified (no product patent, but process patent to be available) New medical uses of known substances (no product patent, but process patent to be available) Derivatives of known medical substances (patent to be available only if enhanced therapeutic efficacy or significant superior properties are established)	s 4(a) s 4(b) s 4(c)

In response to the category one recommendation, partner states have excluded mostly identical subject-matter, the majority of which originates from TRIPS Art. 27(2) and (3).^44^ The implementation of category two exclusions, however, appears contentious, raising crucial issues such as whether excluding natural substances and new uses will conflict with the TRIPS obligation to make patents available in all fields of technology.^45^ Specifically, will it be TRIPS-compliant to exclude product patents in a blanket manner for purified or isolated natural substances and new medical uses of known substances, even when they fulfill all patentability requirements? The prevailing practice among leading WTO members is to offer patent protection to this category of invention once they satisfy some nationally established standards (for instance, being isolated or purified).^46^

However, viewed from the perspective of a region mostly comprised of LDCs, it is possible to argue that this approach is TRIPS-consistent, especially given that EAC LDCs are entitled to the pharmaceutical exemption, which allows them to exclude patents for pharmaceuticals until January 2033.^47^ As the consideration of the transition period obligation shows, most EAC LDCs exclude patent protection for pharmaceutical products, but accept patent applications for pharmaceutical processes.^48^ EAC LDCs should, therefore, embrace this recommendation which offers them additional grounds to exclude certain inventions from being patentable. One challenge to be envisaged for the future though is what the fate of this blanket exclusion would be should EAC LDCs lose their status as LDC or should the Council for TRIPS decline a further extension of the transition period. Perhaps, when the EAC reaches that bridge, it will cross it!

Partner states have implemented the category two recommendations as follows: Uganda, Rwanda, Zanzibar and Burundi exclude natural substances in the exact manner recommended by the EAC;^49^ Kenya (being a developing country) and Tanzania-Mainland (being the only LDC with no patent exemption for pharmaceuticals) neither exclude natural substances nor new medical uses of known substances or derivatives of known medical substances. Similarly, though entitled to do so, Uganda does not exclude “new medical uses” or “derivatives of known medical substances.”

Although other partner states implement the exclusion for new uses and derivatives of known substances, they do not draw a distinction between the two. Thus, Rwanda excludes “known substances for which a new use has been discovered”, but permits patentability if the resulting new use satisfies a strict patentability test.^50^ Both Zanzibar and Burundi take a similar approach, excluding “new uses or forms of known product or process” and “known substances for which a new use has been discovered” respectively.^51^ Unlike Rwanda, these latter partner states do not permit patentability regardless of whether some strict patentability criteria have been fulfilled or not.

Table 6 aggregates the various responses of partner states to the two categories of exclusions recommended by the EAC. The table shows that partner states’ responses to category one exclusions, though similar, are not identical, and include grounds not listed under TRIPS. As argued elsewhere,^52^ the EAC could have adopted a harmonised approach to category one exclusion by iterating subject-matter which partner states must exclude. Table 6 further shows that, in addition to Kenya, Uganda (new medical uses/derivatives of medical substances) and Tanzania-Mainland (both LDCs and therefore entitled to transition-related exclusions) surrender this flexibility.

**Table 6:** Exclusion from patentability (national implementation)

Cat	Kenya	Uganda	Rwanda	Burundi	T-Mainland	Zanzibar
1	Discoveries, scientific theories & mathematical methods Schemes, rules or methods for doing business, performing purely mental acts or playing games Diagnostic, therapeutic or surgical methods for treating human or animal Mere presentation of information Public health related methods of use or uses of any molecule or other substances … used for the prevention or treatment of any disease, provided responsible Minister designates such disease as a serious health hazard or as a life-threatening disease	Discoveries, scientific theories & mathematical methods Schemes, rules or methods for doing business, performing purely mental acts or playing games Diagnostic, therapeutic or surgical methods for treating human or animal Mere presentation of information Plants and animals (other than micro-organisms) & essentially biological processes for producing plants or animals (other than non-biological and micro-biological processes) The human body and all its elements in whole or in part	Discoveries, scientific theories & mathematical methods Schemes, rules or methods for doing business, performing purely mental acts or playing games Diagnostic, therapeutic or surgical methods for treating human or animal Plants and animals (other than micro-organisms) & essentially biological processes for producing plants or animals (other than non-biological and micro-biological processes) Animal and plant varieties Inventions whose commercial use is contrary to public order and morality	Discoveries, scientific theories & mathematical methods Diagnostic, therapeutic or surgical methods for treating human or animal Plants and animals (other than micro-organisms) & essentially biological processes for producing plants or animals (other than non-biological & micro-biological processes) Animal & plant varieties Plans/principles/methods in the field of economic activities or performance of purely mental activities or in games Inventions whose commercial use is contrary to public order & morality	Discoveries, scientific theories & mathematical methods Schemes, rules or methods for doing business, performing purely mental acts or playing games Diagnostic, therapeutic or surgical methods for treating human or animal Plants and animals (other than micro-organisms) & essentially biological processes for producing plants or animals (other than non-biological & micro-biological processes) Mere presentation of information	Discoveries, scientific theories & mathematical methods Schemes, rules or methods for doing business, performing purely mental acts or playing games Diagnostic, therapeutic or surgical methods for treating human or animal Plants and animals (other than micro-organisms) & essentially biological processes for producing plants or animals (other than non-biological & micro-biological processes) Animal & plant varieties The human body & all its elements in whole or in part Inventions whose commercial use is contrary to public order and morality
2	No exclusion for natural substances, new medical uses or derivatives of medical substances	Natural substances excluded No exclusion for new uses/derivative	Natural substances excluded Known substances excluded	Natural substances excluded Known substances excluded	No exclusion for natural substances, new medical uses or derivatives of medical substances	Natural substances excluded New uses or form of known product or process excluded

Another issue worthy of comment from Table 6 is Kenya’s adoption of a controversial exclusion under category one. According to this exclusion, a responsible Minister may exclude from patentability the method of use or uses of *any* molecule or other substances useful for treating or preventing any disease designated by the Minister as a serious health hazard or as being life threatening.^53^ While it is undisputable that WTO members enjoy the prerogative to exclude additional subject-matter, such exclusion must be TRIPS-consistent.^54^ The Kenyan exclusion thus raises the question of whether a total exclusion of such a “molecule” or “substance” from patentability is necessary to achieve the aim of treating or preventing disease(s) designated as life threatening or as constituting a serious health hazard. The answer to this will likely be in the negative since there are other less rights-intrusive avenues within TRIPS that Kenya could invoke to achieve the same outcome – e.g. compulsory licensing or a government use exception.^55^

Overall, there is a need to tidy up these conflicting provisions, as failing to do so may frustrate the purpose intended to be served by the flexibility.

### Research Exception

Like other flexibilities, the research exception – a narrowed TRIPS-sanctioned permission to use a patented invention for research purposes without the patentee’s authorisation^56^ – is a policy tool that the EAC can deploy to set-up an additional non-infringing bubble within which local researchers can continue to hone their research acumen.^57^ Table 7 recaps the regional recommendations on this exception.

**Table 7:** Research exception (regional recommendations)

S/N	Recommendations	EAC Protocol
1	Exempt research on patented invention (commercial or non-commercial)	s 7(1)(1)
2	Limit commercial research to generation of new knowledge	s 7(1)(2)
3	Establish a non-exclusive-compensation-only licensing system for patented research tools	s 7(1)(3)

As usually the case, partner states have responded differently. In compliance with the regional advice, all partner states exempt research activities carried out *on* patented articles for non-commercial purposes,^58^ even though all of them also fail to establish a non-exclusive compensation-only licensing scheme for patented research tools.^59^ Furthermore, only Uganda and Zanzibar exempt research *on* patented articles for commercial purposes, but they both disregard the balancing requirement that such use must be for generating new knowledge.^60^ Partner states’ responses are contained in Table 8.

**Table 8:** Research exception (national implementation)

Partner states	Commercial research	Non-commercial research	Commercial research conditional	Non-exclusive licence (research tools)
Kenya	Not exempted	Exempted	No	Not provided
Zanzibar	Exempted	Exempted	No	Not provided
T-Mainland	Not exempted	Exempted	No	Not provided
Burundi	Not exempted	Exempted	No	Not provided
Uganda	Exempted	Exempted	No	Not provided

Few observations could be made on partner states’ implementation choices. In failing to exempt commercial research, partner states seem to favour the US approach where any inkling of a commercial link defeats the research exception claim.^61^ The reality, though, is that unlike the US, which boasts of sophisticated innovative capacity driven by uniquely accomplished researchers, EAC researchers occupy the lowest level of the innovation curve. EAC researchers therefore stand to benefit from implementing the regional approach (i.e. permitting the research exception for commercial research in limited circumstances).

Secondly, the approach adopted in Uganda and Zanzibar is equally flawed. Both partner states provide a blanket exemption for commercial research, regardless of whether it is intended to advance knowledge or not. In addition to encouraging free riding, this implementation will disadvantage patentees of research tools by exposing them to infringement without compensation. By their nature, research tools are mainly used complementarily in conducting other research, including commercial experimental research.^62^ On the other hand, the first approach is not any better, as it may encourage patentees of research tools to use their exclusive rights to block (e.g. through injunction) any attempt to “research on” patented tools. This practice may jeopardise follow-on research, especially if patentees decline a request for a voluntary licence in respect of these tools. Incorporating a non-exclusive compensation-only licensing scheme, as the region recommends, achieves the right compromise between the two extreme approaches.

### *Bolar* Exception

Unlike the research exception, which any willing WTO member may broadly implement to exempt both infringing commercial and non-commercial research “on” patented articles, the *Bolar* exception applies narrowly to uses reasonably related to obtaining marketing approval for generic or originator pharmaceutical products or, sometimes, products generally.^63^ The exception is of considerable benefit to the pharmaceutical industry and the access-to-medicines campaign because it facilitates early entry of generic pharmaceutical products into the market almost immediately after the patents expire.^64^ EAC recommendations on this exception are outlined in Table 9.

**Table 9:** *Bolar* exception (regional recommendation)

S/N	Recommendations	EAC Protocol
1	Exempt uses reasonably related to obtaining marketing approval	s 7(2)
2	Exemption to cover local and foreign uses	s 7(2)
3	Apply exemption to ‘any product’	s 7(2)

A perusal of national responses shows a contrasting approach. All EAC LDCs (except Tanzania-Mainland), on the one hand, implement the *Bolar* exception as regionally recommended, i.e. by exempting otherwise infringing uses of patented pharmaceutical products prior to patent term expiry provided the purpose of such use is reasonably related to generating dossiers for obtaining marketing authorisation for new or generic products under domestic or foreign law. 65 On the other hand, the IP laws in Kenya and Tanzania-Mainland do not have equivalent provisions, thus rendering infringing any attempt to use, buy or sell patented pharmaceutical products for the purpose of collecting data required for obtaining marketing authorisation at home or abroad.

It is quite paradoxical that Kenya and Tanzania-Mainland, both partner states with moderate pharmaceutical manufacturing capacities and patent protection for pharmaceutical products and processes, ignore a recommendation like this which could theoretically have facilitated early entry of patented pharmaceutical products into the regional market. It is, nevertheless, possible to posit that the non-implementation of a *Bolar* exception in these partner states may not have a negative impact for access since pharmaceutical production capacity in the region is largely focused on basic generic formulations anyway. This notwithstanding, a harmonised approach to implementing this exception could benefit the region in the nearest future when manufacturing capacity eventually peaks (see Table 10 for an overview of partner states’ implementation).

**Table 10:** *Bolar* exception (national implementation)

Partner states	Exception included	Local uses	Foreign uses	Coverage
Kenya	No	No	No	Not applicable
Zanzibar	Yes	Exempted	Exempted	Any product
T-Mainland	No	No	No	Not applicable
Rwanda	Yes	Exempted	Exempted	Any product
Burundi	Yes	Exempted	Exempted	Any product
Uganda	Yes	Exempted	Exempted	Any product

### Test Data Protection

Clinical test data are protected within an IP framework because of the gigantic financial investment which goes into generating them; they are also pivotal to validating the efficacy and safety of proposed pharmaceutical products, without which national regulatory bodies will deny marketing approval.^66^ Two implementation choices are prevalent: a misappropriation regime^67^ (simply protects test data against “unfair commercial use and disclosure”) or a data exclusivity regime (protects any kind of use for certain period).^68^ The EAC recommends the former approach (see Table 11).

**Table 11:** Test data obligation (regional recommendations)

S/N	Recommendations	EAC Protocol
1	Kenya, the only non-LDC partner state, to immediately protect against "unfair commercial use and disclosure" (misappropriation regime)	s 12(1)
2	LDC partner states should postpone compliance to post-transition period	s 12(1)
3	All partner states to allow reliance on submitted data to assess safety and efficacy of subsequent generic applications	s 12(2)

Partner states’ legislative responses could be grouped into three. In group one are Zanzibar and Burundi, both of which adopt a convoluted approach which could be summarised as follows: Where submitted data is in respect of a new chemical entity and involves significant financial and physical efforts to generate, a data exclusivity regime applies up to a maximum period of five years.^69^ A chemical entity is new if it has not been granted marketing approval or marketed anywhere in the world within the period of 18 months following the first marketing approval granted or marketing carried out anywhere in the world.^70^ This data exclusivity regime notwithstanding, the laws in both states permit a reliance on test data by subsequent applications in certain circumstances,^71^ subject to the condition that compensation is paid for the access. The compensation payable is to be agreed between the originator of test data and the party seeking reliance, but, where the two cannot agree, this could be fixed by the marketing approval authority.^72^ Reliance is also permitted where a subsequent applicant demonstrates that they have independent access to the data from a public source.^73^

Group one partner states further permit reliance on test data without compensation in the following circumstances: where reliance is for non-commercial purposes such as where a university or research institution is requested by the government to rely on the data for the purpose of verification; where an applicant seeking reliance undertakes to postpone actual market entry until after the expiry of the exclusivity period; or where reliance is sought for marketing approval purposes in respect of export, accompanied by an undertaking that the products will not be commercialised in Zanzibar or Burundi.^74^ Additionally, where test data relates to non-new chemical entities such as new uses or new indications, both partner states adopt the EAC recommended misappropriation regime under which test data is only protected against “unfair commercial use and disclosure” and regulatory authorities are allowed to rely on submitted test data for approving subsequent applications.^75^

Lastly (perhaps, more noteworthy), the IP laws in both countries postpone the commencement of all the above provisions to a later date. However, while the provision adopted in Zanzibar successfully achieves this purpose, the same cannot be said for Burundi. For instance, in Zanzibar, these provisions will only enter into force after expiry of the country’s transition period (currently January 2033 or any subsequent extensions approved by the TRIPS Council).^76^ Meanwhile, even though the law in Burundi evinces an intention to postpone the commencement date to a later date, the fact that Burundi did not properly implement the transition period obligation to cover future extensions means that these provisions had already been in force in Burundi since January 2016 (the only date recognised in Burundi’s IP law).^77^

Pondering over the convoluted regime favoured in Zanzibar and Burundi, one cannot but wonder what two EAC LDCs, expressly exempted from test data obligations, stand to gain by enacting comprehensive and confusing provisions on test data.^78^ Could the justification be that they both want to encourage technology transfer and foreign direct investment into their domains? It is difficult, however, to see how this could be a valid justification, given that there is no empirical data supporting the hypothesis that a strong IP regime is sufficient in itself to promote investment in, and technology transfer to, LMICs.^79^ What is certain, however, is that the two partner states have committed to adding a test-data monopoly to the already existing patent monopoly immediately the data exclusivity regime enters into force.^80^

Kenya, Tanzania-Mainland and Rwanda are in group two – the trio have no provision on test data protection. The absence of a legal provision in Kenya notwithstanding, an officer of the Poison and Pharmacy Board (PPB) (Kenyan drug regulatory body) told the author that the PPB often relies on dossiers submitted by applicants and that whether separate test data submission would be required would depend on the historical origin of the medical products involved.^81^ For imported products, marketing approval would be granted if the products have already been approved in the country of origin and provided the originating country is among those recognised by PPB for that purpose.^82^ The same rule applies to locally manufactured pharmaceutical products, and since local pharmaceutical firms mainly deal in generic products, the PPB, according to the interviewed officer, considers the historical origin of the product, together with the submitted dossiers to determine whether marketing approval should be granted.^83^ A similar approach applies in Tanzania-Mainland according to interviewed stakeholders.^84^

In the last group is Uganda, which protects test data against unfair commercial use and disclosure using a trade secret law.^85^ While this law is silent on whether reliance on test data by subsequent applicants is permissible, the practice at the National Drug Agency (body responsible for drug regulation) is to allow reliance based on the origin of the pharmaceutical products – similar to the practice in place in Kenya and Tanzania-Mainland.^86^

In sum, while these varied approaches – especially in Zanzibar and Burundi – should generate concerns, the reality is that test data provisions, including the pro-patent owners’ approach embraced in two EAC LDCs, are not likely to have any practical effect in the region for some time. The reason for this is not far-fetched: the leading pharmaceutical firms and importers in the region deal almost entirely in generic products, which do not require the submission of new test data, as already indicated. As a matter of fact, neither Zanzibar nor Burundi has a vibrant pharmaceutical industry, and they both rely heavily on importation within and outside the region.^87^ Tanzania-Mainland, Kenya and Uganda are the hubs of pharmaceutical production for the region and, from conversations with stakeholders, even the capacity of firms in these partner states is limited mainly to basic formulations.^88^ Nevertheless, the adoption of a harmonised misappropriation regime (as regionally recommended) may benefit partner states in the nearest future when regional innovative and manufacturing capacity is expected to have evolved. Table 12 summarises national responses on test data implementation.

**Table 12:** Test data obligation (national implementation)

Partner states	Misappropriation regime	Data exclusivity regime	Reliance permitted	Regime apply post-transition period
Kenya	No provision	No provision	(In practice) Yes	Not Applicable
Zanzibar	Yes	Yes	(If not NCE) Yes	Yes
T-Mainland	No provision	No provision	(In practice) Yes	No provision
Rwanda	No provision	No provision	No provision	No provision
Burundi	Yes	Yes	(If not NCE) Yes	No
Uganda	Yes	No	(In practice) Yes	No

### Disclosure Requirement

The entire patent system is based on a *quid* *pro* *quo* arrangement under which, in return for a promise of limited monopoly rights, patent applicants undertake to fully and sufficiently disclose to the public how to work their inventions.^89^ Thus, information regarding patented inventions becomes publicly available from the publication date, and, while this information cannot be accessed during the patent term for the purpose of commercial replication, it could be explored for incremental research purposes.^90^ Benefitting from this flexibility, however, depends largely on the clarity of national implementation provisions – TRIPS has used the words “sufficiently clear and complete” disclosure.^91^ Any wordings lacking such clarity may be exploited by patentees to game the system by, on the one hand, making insufficient disclosure to obtain patent monopoly, and on the other, keeping essential information on working the invention as trade secrets. The EAC recommendations (Table 13) seem to unwittingly encourage gaming the system.

**Table 13:** Disclosure requirement (regional recommendations)

S/N	Recommendations	EAC Protocol
1	Require disclosure of all modes	s 6(1)
2	Require express indication of "best mode"	s 6(1)

The regional recommendations on this requirement having failed to stress the importance of a “sufficiently clear and complete” disclosure, most partner states fill this lacuna (using different wordings with similar effect) in their implementation. This is in addition to requiring the disclosure of the “best mode” as the EAC recommends. Both Tanzania-Mainland and Zanzibar require disclosure “in a manner sufficiently clear and complete”; patent applicants must also “indicate the best mode for carrying out the invention”.^92^ Kenya and Uganda mandate a disclosure of an invention that is “full, clear, concise and exact … .”^93^ Ugandan law requires the disclosure of “all practicable modes” and the indication of a best mode.^94^ Kenyan law only requires the indication of “the best mode for carrying out the invention” (nothing about “all modes”).^95^

In Rwanda, the preferred language is disclosure in a “manner sufficiently clear, complete and intelligible”, and an additional requirement is that an applicant indicates “the best way” of using the invention.^96^ Lastly, Burundi takes a slightly different approach, requiring on the one hand a disclosure “in a manner that is sufficiently clear and comprehensive”, and on the other, an indication of “at least one embodiment of the invention … .”^97^ In all cases, what amounts to “sufficient and clear disclosure” and/or “best mode” is determined by what is disclosed and indicated at the filing or priority date (where priority is claimed)^98^ – see Table 14 for a summary of national responses.

**Table 14:** Disclosure requirement (national implementation)

Partner states	Sufficiently clear and complete	Indicate all modes	Indicate best mode
Kenya	Yes	No	Yes
Zanzibar	Yes	No	Yes
T-Mainland	Yes	No	Yes
Rwanda	Yes	No	Yes
Burundi	Yes	No	No
Uganda	Yes	Yes	Yes

One identified shortcoming of the EAC approach is its failure to provide a harmonised guide for partner states on implementing the core principle of the disclosure requirement, namely, the need for disclosure to be made in a sufficient and clear manner.^99^ It is, therefore, commendable that partner states redress this shortcoming in their implementation. The need to disclose “all modes” and indicate a “best mode” has also been labelled superfluous because determining what is the best mode is both subjective and difficult.^100^ More challengingly, what is the “best mode” is ultimately ascertained at the time of filing or at priority date, meaning that what appears as the “best mode” at this point would most certainly have changed by the time the patent is granted or litigation instituted.^101^ Since enacting a “best mode” requirement is unlikely to benefit the patent grant process (e.g. by serving invalidity purposes), partner states are better off not implementing it. This will save them the complexity associated with practically implementing this requirement.

### Opposition Procedure

Opposition procedure is another optional TRIPS obligation^102^ which interested WTO members can implement to permit a challenge to patent applications before and/or after grant. Opposition procedure is particularly instrumental for regulating patent evergreening as well as supplementing the knowledge of patent examiners who stand to benefit from the multiple areas of expertise of opponents.^103^ The regionally preferred approach on implementing this obligation is outlined in Table 15.

**Table 15:** Administrative opposition procedure (regional recommendations)

S/N	Recommendations	EAC Protocol
1	Provide for pre-grant opposition procedure	s 3(1)
2	Provide for post-grant opposition procedure	s 3(1)
3	Partner states to determine the grounds for opposition	s 3(1)

Here is what implementation among partner states looks like: three partner states which have existing obligations to offer patent protection either for both pharmaceutical products and processes (Kenya and Tanzania-Mainland) or for pharmaceutical processes only (Rwanda), fail to provide for either a pre-grant or post-grant opposition procedure. These partner states only provide for the revocation or invalidation of patents through the conventional court system.^104^ A similar implementation approach applies in Zanzibar and Burundi, which both make a pre-grant opposition procedure available. In Zanzibar, an application opposing patent grant must be brought after the publication of the patent application but before grant, whereas in Burundi, such application must be brought within 90 days of the publication of patent applications.^105^ This leaves Uganda as the only partner state which follows the EAC proposition of combining both pre- and post-grant opposition procedures. As in Burundi, Uganda requires a pre-grant opposition application to be brought within 90 days, while a post-grant application must be filed within one year.^106^

As can be seen from Table 16, the grounds upon which opposition (pre- and/or post) applications may be brought differ from one partner state to another.^107^ This raises two concerns: (1) the regional approach is deficient by failing to harmonise opposition grounds for partner states;^108^ and (2) regrettably, partner states for which these recommendations could have made a difference have not implemented them. Regarding the latter concern, out of the three partner states with moderate pharmaceutical manufacturing capacity (Kenya, Tanzania, and Uganda), only Uganda has followed the EAC approach (pre- and post-grant opposition procedures).^109^ The irony in this though is that Uganda currently has no obligation to offer patent protection to pharmaceutical products and cannot therefore benefit from the implementation of this approach since there are no patent applications to oppose.^110^ The reality in Zanzibar and Burundi is even worse with no significant pharmaceutical manufacturing capacity in their states which could have benefitted from the provision. Moreover, combining both pre- and post-grant opposition procedures has been rightly criticised for its many challenges, including causing inordinate delay.^111^

**Table 16:** Opposition procedure (national implementation)

Partner states	Pre-grant	Post-grant	Revocation or invalidation	Grounds
Kenya	No	No	Yes	Patent granted to wrong inventor Infringement of earlier patent right by patentee Failure to meet patentability criteria Insufficient disclosure Non-stipulation of best mode Material misrepresentation in application process
Zanzibar	Yes	No	Yes	Failure to meet formality requirements Failure to meet patentability criteria Invention excluded from patentability
T-Mainland	No	No	Yes	Failure to meet patentability criteria, Invention excluded from patentability Insufficient disclosure Non-stipulation of best mode Patent granted to wrong inventor
Rwanda	No	No	Yes	Claim-related issues Failure to meet patentability criteria Invention excluded from patentability Insufficient disclosure Non-stipulation of best mode
Burundi	Yes	No	Yes	No grounds are expressly stipulated. Law only provides: "such opposition shall indicate … the arguments and evidence put forward by the opposing party to prevent the grant of the patent".
Uganda	Yes	Yes	Yes	Failure to meet patentability criteria Invention excluded from patentability Failure to comply with formality requirements
Kenya	No	No	Yes	Patent granted to wrong inventor Infringement of earlier patent right by patentee Failure to meet patentability criteria Insufficient disclosure Non-stipulation of best mode Material misrepresentation in application process.
Zanzibar	Yes	No	Yes	Failure to meet formality requirements Failure to meet patentability criteria Invention excluded from patentability

### Exhaustion Regime

TRIPS Art. 6 makes it crystal clear that WTO members are in absolute control vis-à-vis choice of exhaustion regime. Exhaustion (of patent rights) defines the point when a patentee’s right of first sale becomes “*exhausted*” and subsequent non-authorised sales by others stop being infringing – provided of course that the original sale is by the patentee or their authorised agent.^112^ Three regimes of exhaustion are recognised: national, regional, and international.^113^ Whether a country can invoke parallel importation to import pharmaceuticals from other assumedly cheaper markets depends largely on the regime of exhaustion in place in that country – both regional and international exhaustion regimes allow parallel importation with the main difference in the scope of the destination market, while national exhaustion without more prohibits parallel importation.^114^ As is to be expected, the EAC recommends an international exhaustion regime as per Table 17 below.

**Table 17:** Exhaustion regime (regional recommendation)

S/N	Recommendation	EAC Protocol
1	Provide for international exhaustion regime	s 3(1)

Three implementation groups could be identified from partner states’ patent laws. In the first group are Kenya, Uganda and Zanzibar, all of whom follow the EAC advice by exempting “acts in respect of articles which have been put on the market in Kenya [Uganda or Zanzibar] or in any other country or imported into Kenya [Uganda or Zanzibar].”^115^ In these partner states, the use of parallel importation is made available *unconditionally*.

Burundi is in the second group: “acts relating to goods placed on sale in Burundi or in any other country by the patent holder or with his consent …” are exempted from infringement.^116^ This provision is indicative of an international exhaustion regime and, by implication, availability of parallel importation. Unlike group one partner states though, the use of parallel importation in Burundi is heavily regulated as follows: only the Minister responsible for trade is empowered to authorise the use of parallel importation, a power the Minister can exercise *suo* *motu* or at the request of an interested party.^117^ A request from an interested party will only be considered if it relates to products which are not available in Burundi, or are available in insufficient quality or quantity to meet local needs or if the price charged locally is exorbitant or if such request is justified in the interest of the public.^118^ Furthermore, the Minister may revoke (for failure to meet the goal of the grant) or cancel (where conditions justifying the grant no longer exist) already authorised parallel importation.^119^

Rwanda and Tanzania-Mainland are in group three. Both apply a national exhaustion regime which generally precludes parallel importation.^120^ Both partner states, however, deviate from the general rule by permitting the use of parallel importation in exceptional circumstances: in Tanzania-Mainland, parallel importation may be authorised for any drug if the designated Authority believes it is in the public interest to do so.^121^ The procedure in Rwanda is more elaborate but identical to that in place in Burundi – especially regarding the grounds for requesting, revoking and cancelling an application for parallel importation.^122^ These responses are tabulated in Table 18 below.

**Table 18:** Exhaustion regime (national implementation)

Partner states	Exhaustion regime	Parallel importation	Conditions attached
Kenya	International	Yes	None
Zanzibar	International	Yes	None
T-Mainland	National	Yes	Designated Authority must authorise use Use must be in the public interest
Rwanda	National	Yes	Can only be used for drugs Not available in Rwanda or Available in Rwanda in poor standard or Available in Rwanda in insufficient quantities or Available in Rwanda but price unfair May be revoked if not used for justified purpose May be cancelled where no longer needed
Burundi	International	Yes	Can only be used for drugs Not available in Burundi or Available in Burundi in poor standard or Available in Burundi in insufficient quantities or Available in Burundi but price unfair May be revoked if not used for justified purpose May be cancelled where no longer needed
Uganda	International	Yes	None

A few points can be deduced from the data in Table 18. First, the approach in Burundi, Rwanda and Tanzania-Mainland, though not in complete compliance with the regional recommendation, aligns with the author’s arguments elsewhere that partner states should implement parallel importation in a manner which convincingly assures multinational pharmaceutical firms of protection against re-exportation – this is expected to encourage these firms to price-discriminate for patented pharmaceutical products sold into the region.^123^ The Rwandan approach may be particularly singled out as a perfect solution to one of the criticisms levelled against the EAC approach (i.e. promoting absolute use of parallel importation conflicts with the regional objective of seeking to enhance regional pharmaceutical production capacity).^124^ This will unquestionably be addressed by the provisions in Rwanda that parallel importation should only be used in respect of drugs for which there is no or insufficient local manufacturing capacity. While Burundi has similar limiting provisions, the implementation of an international exhaustion regime robs those provisions of any pragmatic efficacy. This is because the Burundian provisions only limit the national use of parallel importation, they do not preclude international importers from coming into Burundi to re-export cheaper medical products which may have entered Burundi through parallel importation.

The above responses may be contrasted with those of Kenya, Zanzibar and Uganda, where no limit is imposed on the use of parallel importation. It is doubtful that these countries can derive any practical benefit from an unregulated use of parallel importation for reasons canvassed elsewhere – a regional exhaustion regime with adequate regulatory framework to prevent re-exportation appears to offer a better solution.^125^

### Compulsory Licensing

TRIPS Art. 31 (as amended by Art. 31^bis^) enumerates the conditions with which WTO members must comply to use a compulsory licence either for local production or import/export.^126^ Members with sufficient local manufacturing capacity can rely on the national equivalence of these provisions to issue a compulsory licence to a local pharmaceutical firm authorising it to manufacture urgently needed medicines. In the same vein, WTO members with no (or with insufficient) pharmaceutical manufacturing capacity are now able, relying on the provisions of TRIPS Art. 31^bis^, to issue a compulsory licence which a recipient local pharmaceutical firm can use to import essential medicines from an overseas pharmaceutical firm, the latter firm having also been issued with a compulsory licence by its home government – this has been tagged compulsory-licence-for-export.^127^ This flexibility has particularly been promoted as capable of addressing the access-to-medicines conundrum ravaging several LMICs. It is therefore important to analyse the comprehensive recommendations of the EAC on implementing this all-important flexibility – see Table 19.

**Table 19:** Compulsory licence obligation (regional recommendations)

S/N		Recommendations	EAC Protocol
1	Grounds	National emergency or other situations of extreme urgency or public non-commercials use To remedy anti-competitive practices and abuse of exclusive rights Failure to satisfy local demand (in terms of quality, quantities or fair price) Public interest Interdependent patents To give effect to TRIPS Art 31^bis^	s 8(1)
2	Compulsory licence (CL) for export	Draft comprehensive guidelines or regulations to implement TRIPS Art 31^bis^ Implement both as eligible importing country and eligible exporting country	s 8(3)
3	Prior negotiation	Must be completed within 90 days Waive in cases of national emergency, other situations of extreme urgency, for public non-commercial use or where CL is issued to remedy anti-competitive practices	s 8(2)
4	Compensation	Royalty must not exceed 4% of turnover Factor anti-competitive practices in calculation Where CL is for export, consider economic value of use to eligible importing country Waive compensation where CL is issued for export, and the eligible exporting country has already paid compensation to patentholder.	s 8(3)
5	Exclude injunction	Provisions may be made for further appeal to court But limit rightsholder’s remedy to recovery of adequate compensation	s 8(4)
6	Competent authority	Administrative entity should be preferred	s 8(5)

Three types of compulsory licence could be discerned from Table 19: use by individuals, government use, and use for export. A perusal of partner states’ legislation shows significant compliance with regional recommendations on the first two,^128^ but, as later analysis will show, not so much for the compulsory-licence-for-export regime.^129^ Detailed comparative analysis of partner states’ implementation of each of the above themes follows in Tables 20, 21, 22, 23, 24, 25 below.

**Table 20:** National implementation of the “grounds for granting CL” requirement

Kenya	Tanzania-Mainland	Uganda	Rwanda	Burundi	Zanzibar
Non- or insufficient working of patent (4 years after filing or 3 years after grant)	Non- or insufficient working of patent (4 years after filing or 3 years after grant)	Non- or insufficient working of patent (4 years after filing or 3 years after grant)	Non- or insufficient working of patent (4 years after filing or 3 years after grant)	Non- or insufficient working of patent (4 years after filing or 3 years after grant)	Non- or insufficient working of patent (4 years after filing or 3 years after grant)
Interdependent patents	Interdependent patents	Interdependent patents	Insufficient quality or quantities or higher prices	Insufficient quality or quantities or higher prices	Insufficient quality or quantities or higher prices
National security, nutrition, health, environmental conservation	National security, nutrition, public health	National security, nutrition, health, environmental conservation	Interdependent patents	Interdependent patents	Interdependent patents
*Development* *of* *vital* *sector* *of* *national* *economy*	*Development* *of* *vital* *sector* *of* *national* *economy*	*Development* *of* *vital* *sector* *of* *national* *economy*	National security, public health, environmental protection	National security, nutrition, health, environmental conservation	National security, nutrition, health, environmental conservation
Anti-competitive practices		CL for export	Anti-competitive practices	CL for export	CL for export
				*Development* *of* *vital* *sector* *of* *national* *economy*	*Development* *of* *vital* *sector* *of* *national* *economy*
				Anti-competitive practices	Anti-competitive practices

**Table 21:** National implementation of the “prior negotiation” requirement

Kenya	Tanzania-mainland	Uganda	Rwanda	Burundi	Zanzibar
Implemented, but no time-limit specified	Implemented, but no time-limit specified	Implemented, but no time-limit specified	Implemented, but no time-limit specified	Implemented	Implemented
Can be waived	Can be waived	Can be waived	Can be waived	Must be concluded within 6 months	Must be concluded within 90 days
				Can be waived	Can be waived

**Table 22:** National implementation of the “compensation” requirement

Kenya	Tanzania-mainland	Uganda	Rwanda	Burundi	Zanzibar
Implemented, but no percentage specified	Implemented, but no percentage specified	Implemented, but no percentage specified	Implemented, but no percentage specified	Implemented, but no percentage specified	Implemented with a benchmark of 4% royalty
					To be waived in cases of CL for export where payment has been made in the exporting country

**Table 23:** National implementation of the “injunctive relief” requirement

Kenya	Tanzania-Mainland	Uganda	Rwanda	Burundi	Zanzibar
Appeal lies to Tribunal or court	Implemented for government use – appeal shall not operate as a stay	Appeal lies to the competent tribunal	Appeal lies to the competent tribunal	Appeal lies to court	Implemented, appeal shall not operate as a stay
Law silent on whether injunctive relief is prohibited	Applicant’s only remedy is compensation (for government use only)	Law silent on whether injunctive relief is prohibited	Law silent on whether injunctive relief is prohibited	Appeal operates as a stay of execution	Applicant’s only remedy is compensation
	Law silent on individual use of CL

**Table 24:** National implementation of the “competent authority” requirement

Kenya	Tanzania-MAINLAND	Uganda	Rwanda	Burundi	Zanzibar
Tribunal (for individual applicants)	Minister (for government use)	Minister	Minister	Minister (for government use)	Minister
Minister (for government use)	Court (for individual use)			Court (for individual applicants)

**Table 25:** National implementation of the compulsory-licence-for-export regime

Kenya	Tanzania-mainland	Uganda	Rwanda	Burundi	Zanzibar
Not implemented	Not implemented	Made limited reference to CL for export under exception to patent rights	Not implemented	WTO General Council Decision of 30 Aug 2003 referenced	Indirect reference made to using it as importing country
		Provide for use as exporting country		Provide for use as exporting country	Provide for use as exporting country

 Table 20 shows substantial similarity in implemented grounds for granting compulsory licences among partner states, even though some grounds not regionally recommended are also included in some cases. One of these grounds (the development of a vital sector of the national economy)^130^ is, however, controversial. This is because the right of WTO members to derogate from TRIPS obligations in favour of national interests is subject to the umbrella condition that such derogation must be TRIPS-consistent.^131^ It is, thus, difficult to see how a blanket use of a compulsory licence for industrial purposes (as proposed here) can satisfy the TRIPS-consistency test.^132^

According to the regional recommendation, negotiations for a voluntary licence (which must not exceed 90 days) between patentees and the person seeking a compulsory licence must have failed before the latter can bring an application for a compulsory licence. Comparing partner states’ responses (see Table 21), only Zanzibar implements this to the letter.^133^ In Burundi, a time stipulation of six months (as opposed to 90 days) is legislated.^134^ As for other partner states, while the need for prior negotiation is recognised, no specific timeframe is mentioned.^135^ The importance of capping the negotiation timeframe includes precluding an unscrupulous patentee from exploiting the uncertainty to unnecessarily delay the negotiation process.

Table 22 on “compensation” reveals that while all partner states make provisions for payment of compensation, only Zanzibar includes the regionally specified royalty percentage.^136^ This recommendation could serve a dual purpose: first, it assures patentees that, even when their patent rights are exploited without their consent, they will still be compensated; second, implementing the recommended percentage (4%) puts a ceiling on the royalty rate patentees can claim as adequate compensation.

The national implementation of “injunctive relief” is represented in  Table 23.

As can be gleaned from this table, only Zanzibar expressly provides that an appeal to the court shall not act as a stay of execution and that an appellant patent owner will only be entitled to a review of the compensation payable.^137^ In Tanzania-Mainland, a similar provision exists for an appeal in respect of government uses, but not in respect of individual applicants.^138^ In Kenya, Uganda and Rwanda, the applicable laws are silent on injunctive relief, whereas in Burundi the opposite is the case – an appeal operates as a stay of execution.^139^ Since compulsory licences are granted when extremely necessary, excluding injunctive relief or not allowing an appeal to operate as a stay is the only way to guarantee that the purpose of a compulsory licence application is not defeated.

Table 24 recommends the use of an administrative as opposed to a judicial granting authority. This recommendation is apparently aimed at avoiding the delay associated with the conventional court system. However, only few partner states implement this recommendation. In Kenya, Tanzania-Mainland and Burundi, “government use” applications are considered by administrative bodies, while applications from individuals go through conventional courts.^140^ Using the conventional court system or allowing appeals to constitute a stay, contrary to the regional recommendation, will defeat the rationale for the existence of this flexibility.

Finally, Table 25 relates to compulsory licence for export. This became imperative as a means of assuaging the concerns of WTO members with no pharmaceutical manufacturing capacity – something required for the domestic use of a compulsory licence.^141^ The compulsory-licence-for-export regime has a special appeal to regional economic communities (RECs) like the EAC as they are allowed to use the regime to bulk-procure essential medicines (including active pharmaceutical ingredients) from overseas for subsequent distribution among their members – provided of course that such REC has 50% LDC membership.^142^ In addition, unlike developing and developed WTO members, there is a presumption of insufficient manufacturing capacity in favour of LDCs, making it easier for them to use the regime.^143^ This, therefore, seems like a perfect flexibility for EAC partner states to implement – being a REC of seven partner states with six LDCs. This hypothetically means Kenya, the only EAC developing country, can front the use of the compulsory-licence-for-export regime to bulk-procure patented active pharmaceutical ingredients and/or essential medicines for sharing among partner states. This can potentially solve the problem of economies of scale which remains the main cause of the high cost of medications throughout the region.

Unfortunately, however, as seen in Table 25, Zanzibar is the only partner state with the closest provisions to those recommended by the region.^144^ Burundi^145^ and Uganda^146^ then follow, with indirect references to the regime. This leaves Kenya, Tanzania-Mainland, and Rwanda with no provision on the regime. While the failure to implement or fully implement this regime has been rightly criticised, it is equally important to recognise that, although the regime seems like a perfect fit for solving the access conundrum, it is by no means a silver bullet.^147^ The use of the regime by Rwanda to import antiretrovirals from Canada in 2008 particularly opens the regime to well-founded criticisms, predominantly focused on the convoluted processes that must be complied with before using the regime.^148^ This notwithstanding, EAC partner states stand to gain more by implementing the regime. This is particularly more so since some of its criticisms have now been addressed by the recent clarifications supplied in the Ministerial Decision of June 2022 on using TRIPS art 31bis and compulsory licences generally – this Decision was handed down in response to the access-to-medicines challenges raised by the COVID-19 pandemic.^149^ Additionally, a collective use of the regime as a region will avoid the quantity-related challenge identified in its use by Rwanda.^150^

## National Implementation and TRIPS-Plus Obligations

This section briefly discusses the surprising enactment of TRIPS-plus obligations by some partner states in contradiction to an explicit regional counsel against doing so.^151^ The two dubious obligations are patent linkage and patent term adjustment: the former describes the practice of coupling the grant of marketing authorisation for a new medical product to the patent status of that product (where it is found to be under patent, the applicant must demonstrate they have the patentee’s authorisation to register the product, otherwise the application for approval will fail),^152^ while the latter applies to compensate patent applicants/patentees for excessive administrative delays experienced during the patent or marketing approval grant process.^153^ Both obligations are TRIPS-plus because they exceed the minimum standards required under TRIPS.^154^

As mentioned above, the regional Protocol expressly advises partner states against implementing a patent linkage regime; however, reading through partner states’ legislation, only Zanzibar and Burundi implement this recommendation.^155^ Though not expressly excluded in Rwanda and Tanzania-Mainland, representatives of pharmaceutical firms (Tanzania-Mainland) and the marketing approval authority (Rwanda) told the author that a linkage regime was not applied in practice.^156^ Uganda, on the other hand, explicitly provides for a patent-linkage regime; thus, an application for the registration of a drug can only be brought by a patent owner or someone authorised by them.^157^ Finally, Kenya likewise has no statutory provision for patent linkage; however, according to the interviewed PPB officer, the patent status of a drug often plays a pivotal role in determining whether an application to register a drug will be approved or not.^158^

Patent term adjustment has been implemented in two partner states (Burundi and Zanzibar) where the original patent term is 20 years from filing date.^159^ In Zanzibar, the Registrar has a discretionary power to adjust patent term beyond 20 years if it takes more than four years from filing date for the patent to be granted.^160^ The Registrar’s power is, however, limited in two respects: one, extension only compensates for the period of time spent in excess of four years post-filing; and two, the exercise of this power is not automatic (a patentee/patent applicant must apply for it).^161^ A slightly different rule applies in Burundi: should the patent grant process exceed four years post-filing date, upon grant, the patent term is automatically adjusted/extended to cover the entire period of the administrative delay – inclusive of the four years.^162^ Table 26 sets out the state of TRIPS-plus obligations among EAC partner states.

**Table 26:** TRIPS-plus obligations in partner states

Partner states	Patent linkage	Patent term adjustment
Kenya	Yes (in practice)	No
Zanzibar	No (expressly excluded)	Yes (only extra time over 4 years post-filling)
T-Mainland	No (in practice)	No
Rwanda	No (in practice)	No
Burundi	No (expressly excluded)	Yes (4 years + extra time post-filling date)
Uganda	Yes (statutorily required)	No

It is quite difficult to fathom what the rationale is for incorporating TRIPS-plus obligations by partner states in a region which seeks to optimise the use of TRIPS flexibilities in strengthening pharmaceutical manufacturing capacity and improving access to medicines. Supposedly, partner states must have known that adopting these excessive, patentee-friendly obligations will complicate the achievement of the regional objective. Take patent linkage for example: not only will it empower a patentee to challenge a drug registration application on the ground that the products covered by the application infringe patent rights, but also such intervention puts the consideration of the application on hold until the patent challenge has been resolved. Without a linkage system, the application for marketing approval and a challenge to patent rights are treated as two distinct events, so that the occurrence of one does not automatically affect the other. Patent term adjustment also constitutes a clog in the wheel of progress towards the regional goal since its enactment adds an extra layer of monopoly to patentees, thereby potentially delaying the early entry of generic drugs into the regional markets.^163^

## Concluding Remarks

Given the relatively small population of each EAC partner state,^164^ coupled with the situation of an almost inexistent pharmaceutical manufacturing capacity in the region, the decision to act collectively in search of a common solution to the region’s access-to-medicines conundrum cannot be faulted. For one, a regional approach allows partner states to leverage their collective population numbers for the purpose of economies of scale, with the expected end-goal of reduced production costs for the pharmaceutical products procured in the region. More importantly, this approach could, in the long run, facilitate the consolidation and harnessing of pharmaceutical manufacturing capacity across the region, with Kenya, Uganda and Tanzania (Mainland), all of which already possess moderate manufacturing capacities, taking the lead. The identified benefits of a regional approach notwithstanding, this paper has shown that even the best collectively adopted policy framework is bound to fail if its contents are not duly and coherently implemented. Hence, the paper’s focus on a comparative analysis of regional recommendations on optimising TRIPS flexibilities with actual implementation in individual partner states.

Focusing on ten TRIPS obligations, the paper found that not only have partner states failed to implement all the regional recommendations, but also there is a lack of coherence in the implementation of some crucial obligations. Specific examples discussed above include Tanzania-Mainland, which offers patent protection for pharmaceutical products when it could have deployed the transition period flexibility to exclude such protection; Burundi and Zanzibar, which both enact a suspended data exclusivity regime, thereby adding another layer of exclusivity to patentees’ rights; and Kenya, Tanzania-Mainland and Rwanda, which have failed to legislate any opposition procedure. These dark spots of incoherent implementation could potentially have a defeating effect on the overall regional goal of enhancing pharmaceutical manufacturing capacity and ultimately boosting access to essential medicines. Equally defeating, according to the paper’s finding, is the implementation of two TRIPS-plus obligations – patent linkage in Kenya and Uganda and patent term adjustment in Zanzibar and Burundi. Rather than facilitating the early entry of generic pharmaceutical products into the EAC market as envisioned under the regional policy, these obligations will delay such entry and by implication deprive EAC populations of the opportunity for timely access to essential pharmaceutical products.

All hope is, however, not lost! The above analysis also revealed that, in addition to many regional recommendations which are implemented to the letter by partner states, some of the non-complying national implementation options are more suited to achieving the regional goal of improved access than the original regional recommendations. One example is the favoured implementation approach on the disclosure requirement: while the regional recommendation omitted the need for disclosure to be sufficiently clear and complete, the legislation in partner states fill in this salient requirement. Many EAC LDCs have also disregarded the regional recommendation against offering patent protection for pharmaceutical processes – an approach that the paper argued could help improve the research skill of local innovators. Yet another instance is the preferred definition of a person skilled in the art among partner states. Contrary to the regional recommendation (highly skilled), many partner states define this person as someone of ordinary skill. Lastly, some partner states’ approach to the exhaustion regime deviates from the regional recommendation by opting for national (instead of international) exhaustion, with the possibility of using parallel importation in exceptional circumstances. As contended in the paper, this is more aligned to the regional goal of enhancing pharmaceutical manufacturing capacity because it ensures that parallel importation will only be used sparingly.

Going forward, for this and other regional policies on improving access to medicines to be effective, partner states must show more commitment in implementing obligations willingly assumed when acting as part of the region. This is of utmost importance in view of the centrality of coherent implementation to the success of the bigger regional plan, since the EAC framework on TRIPS flexibilities is just a piece in a bigger regional policy puzzle intended to achieve a gradual self-sufficiency in pharmaceutical production. As for the bigger policy puzzle, this is laid out in the EAC Pharmaceutical Manufacturing Plan of Action^165^ and encompasses partner states adopting a common approach to implementing TRIPS flexibilities, drug regulations and pooled procurement.^166^

## Footnotes

1 Leading examples include donations from the US President’s Emergency Plan for AIDS Relief, the Clinton Health Access Initiative, and the Global Fund to Fight AIDS, Tuberculosis and Malaria.

2 In Africa, regional economic communities like the EAC, the Economic Community of West African States (ECOWAS) and the Common Market for Eastern and Southern Africa (COMESA) have adopted various policy frameworks to address the access conundrum in their domains: *see* Owoeye (2019).

3 *See* the East African Community Regional Intellectual Property Policy on the Utilisation of Public Health-Related WTO-TRIPS Flexibilities and the Approximation of National Intellectual Property Legislation (EAC Secretariat) 2013; the East African Community Health Protocol on Public Health Related WTO-TRIPS Flexibilities (EAC Secretariat) 2013.

4 *See* the East African Community Regional Pharmaceutical Manufacturing Plan of Action 2017–2027 (EAC Secretariat).

5 *See* the East African Community, African Medicines Regulatory Harmonisation Programme (AMRH): Launch of the East African Community Medicines Registration Harmonization Initiative Concept Note (EAC Secretariat) 2012.

6 *See* the EAC Policy on Anti-Counterfeiting, Anti-Piracy and other IPR Violations (EAC Secretariat) 2009.

7 South Sudan is currently going through the procedure for acceding to the WTO: *see* WTO, Accession: South Sudan available at https://www.wto.org/english/thewto_e/acc_e/a1_south_sudan_e.htm (accessed 20 December 2022).

8 *See* the Marrakesh Agreement Establishing the World Trade Organisation, opened for signature 15 April 1994, 1869 UNTS 299 (entered into force 1 January 1995) annex 1C (Agreement on Trade-Related Aspects of Intellectual Property Rights).

9 The EAC Protocol on TRIPS Flexibilities clothes the recommendations under the EAC Policy on TRIPS Flexibilities with legality; however, for the Protocol to become binding, all partner states must have ratified it. Unfortunately, this ratification has never happened.

10 *See* Olatunji (2023) (forthcoming in the Journal of African Law).

11 *See* Olatunji (2020), chapter one.

12 *Ibid*.

13 *See* the Constitution of the United Republic of Tanzania Act No. 15 of 1984, Arts. 4(3), 62(1), 64(2) and 106(1)–(3).

14 One year for developed countries; 5 years for developing countries and countries in transition from a centrally planned economy to a free-enterprise economy; 10 years for developing countries that will be extending product patent protection to new areas of technology; and 10 years for LDCs (subject to further extensions) – *see* TRIPS Arts. 65 and 66.

15 TRIPS Art. 66(1).

16 *See* Extension of the Transition Period under Art. 66.1 of the TRIPS Agreement for Least Developed Country Members for certain Obligations with respect to Pharmaceutical Products, Decision of 6 November 2015 (IP/C/73).

17 *See* The Industrial Property Act, Act 3 of 2014 (Uganda) (IPA Uganda) Sec. 8(3)(f); Industrial Property Act No. 4 of 2008 (Zanzibar) (IPA Zanzibar) Sec. 3(1)(x).

18 *Ibid*.

19 IPA Uganda Sec. 28(13)–(14); IPA Zanzibar Sec. 10(8).

20 The Protection of Intellectual Property Law No. 31/2009 (Rwanda) (Rwandan Law No. 31/2009) Art. 18(8); Law Relating to Industrial Property, Law No. 1/13 of 2009 (Burundi) (Burundian Law No. 1/13) Art. 17.

21 Rwandan Law No. 31/2009 (n 20) Art. 18(8).

22 Burundian Law No. 1/13 (n 20) Art. 17.

23 EAC Policy on TRIPS Flexibilities, p. 13.

24 *See* generally Mitsumori (2018).

25 TRIPS Art. 66(1).

26 Submission based on interview responses from representatives of six pharmaceutical companies in Kenya, Uganda and Tanzania: Interviews with Representatives of Six Pharmaceutical Firms (Olugbenga Olatunji, Nairobi, Kampala and Dar es Salam, April to June 2018).

27 *See* TRIPS Arts. 27(2)–(3) and 29.

28 Olatunji *supra* note 10.

29 *Ibid*.

30 *See* The Industrial Property Act (Kenya) No. 3 of 2001 (IPA Kenya) Sec. 23(2); IPA Uganda Sec. 10(2); Rwandan Law No. 31/2009 Art. 15; The Patents (Registration) Act (Tanzania-Mainland) Chapter 217 of 1995 (Patents Act Tanzania-Mainland) Sec. 9(2); Burundian Law No. 1/13 Art. 4; IPA Zanzibar Sec. 4(1)(b).

31 IPA Kenya Sec. 25; IPA Uganda Sec. 12; Rwandan Law No. 31/2009 Art. 17; Patents Act Tanzania-Mainland Sec. 11; Burundian Law No. 1/13 Art. 7; IPA Zanzibar Sec. 4(4).

32 IPA Kenya Sec. 24.

33 IPA Uganda Sec. 11.

34 Rwandan Law No. 31/2009 Art. 16.

35 Patents Act Tanzania-Mainland Sec. 10.

36 Burundian Law No. 1/13 Art. 6.

37 IPA Zanzibar Sec. 4(3).

38 Nielsen and Nicol (2019), pp. 344–346.

39 Olatunji *supra* note 10.

40 *Ibid*.

41 Kenya Industrial Property Institute, Guideline for the Examination of Patents, Utility Models and Industrial Designs (2007) G 6.33.3.

42 TRIPS Art. 27(2)–(3).

43 *See* TRIPS Arts. 7 and 8 for instance.

44 IPA Kenya Sec. 21(3); IPA Uganda Sec. 8(3); Rwandan Law No. 31/2009 Art. 18; Patents Act Tanzania-Mainland Sec. 7(2); Burundian Law No. 1/13 Art. 17; IPA Zanzibar Sec. 3(1).

45 TRIPS Art. 27(1).

46 *See* Dreyfuss et al. (2018); Nicol et al. (2019); Cockbain and Sterckx (2012), p. 367.

47 Decision of 6 November 2015 *supra* note 16.

48 *See* Section 2.1 above.

49 IPA Uganda Sec. 8(3)(g); Rwandan Law No. 31/2009 Art. 18(4); Burundian Law No. 1/13 Art. 17; IPA Zanzibar Sec. 3(1)(iv).

50 Rwandan Law No. 31/2009 Art. 18(5).

51 IPA Zanzibar Sec. 3(1)(v); Burundian Law No. 1/13 Art. 17.

52 Olatunji *supra* note 10.

53 IPA Kenya Sec. 21(3)(e).

54 Olatunji *supra* note 10.

55 *See* IPA Kenya Secs. 72–77, 80.

56 *See* TRIPS Art. 30; this is discussed at length in Olatunji *supra* note 10.

57 It is important to note that the research exception only exempts otherwise infringing uses if they are carried out “on” and not “with” the patented research tools: *see* generally Jaenichen and Pitz (2015).

58 IPA Kenya Sec. 58(1); IPA Uganda Sec. 44(a); Rwandan Law No. 31/2009 Art. 41(2); Patents Act Tanzania-Mainland Sec. 38(1); Burundian Law No. 1/13 Art. 57(3); IPA Zanzibar Sec. 12(4)(a)(iii).

59 EAC Protocol on TRIPS Flexibilities Sec. 7(1).

60 IPA Uganda Sec. 44(a); IPA Zanzibar Sec. 12(4)(a)(iii).

61 For a comprehensive discussion of the US approach, *see* Rowe (2006); Weschler (2004); Russo and Johnson (2015); Dreyfuss (2016).

62 Nielsen and Nicol *supra* note 38.

63 Russo and Johnson *supra* note 61

64 *Ibid*; also Correa 2015, p. 5.

65 IPA Uganda Sec. 44(c); Rwandan Law No. 31/2009 Art. 41(3); Burundian Law No. 1/13 Art. 57(5); IPA Zanzibar Sec. 12(4)(a)(v).

66 *See* generally Skillington and Solovy (2003).

67 Olatunji *supra* note 10.

68 *Ibid*.

69 IPA Zanzibar Sec. 72(5)(a)–(b); Burundian Law No. 1/13 Arts. 375–376.

70 IPA Zanzibar Sec. 72(5)(f); Burundian Law No. 1/13 Art. 380.

71 These circumstances include where obtaining original data has caused suffering to human and animal, or in cases of extreme urgency, or in cases of failure to commercialise the product within a reasonable time after grant of approval, or where compulsory licensing has been granted: *see* IPA Zanzibar Sec. 72(5)(c); Burundian Law No. 1/13 Art. 377.

72 *See* IPA Zanzibar Sec. 72(5)(c); Burundian Law No. 1/13 Art. 377.

73 IPA Zanzibar Sec. 72(5)(e); Burundian Law No. 1/13 Art. 379.

74 IPA Zanzibar Sec. 72(5)(g); Burundian Law No. 1/13 Art. 381.

75 IPA Zanzibar Sec. 72(5)(f); Burundian Law No. 1/13 Art. 380.

76 IPA Zanzibar Sec. 72(5)(h).

77 Burundian Law No. 1/13 Art. 382.

78 One possible explanation may be that these provisions are externally influenced by the World Intellectual Property Organisation, a body responsible for providing “technical assistance” to LDCs in drafting their IP laws: *see* Deere-Birkbeck and Marchant (2011), pp. 109–110.

79 *See* Hall (2014), chapter two; Maskus and Reichman (2004), p. 279; Branstetter (2004).

80 IPA Zanzibar Sec. 72(5)(h); Burundian Law No. 1/13 Art. 382.

81 Interview: Poison and Pharmacy Board of Kenya (Olugbenga Olatunji, Nairobi, Kenya, 18 April 2018).

82 *Ibid*.

83 *Ibid*.

84 Although the contact management officer at the Tanzania Food and Drug Authority declined to participate in an interview session with the author, representatives of two pharmaceutical firms which participated in the interviews told the researcher that the Kenyan rule on test data also applied in Tanzania – Interviews: Mansoor Daya Pharmaceutical Ltd (Tanzania) (Olugbenga Olatunji, Dar es salaam, Tanzania, 28 May 2018); ZENUFA Laboratories (Olugbenga Olatunji, Dar es salaam, Tanzania, 2 June 2018).

85 *See* Trade Secrets Protection (Uganda) Act of 2009, Sec. 11.

86 Interview: National Drugs Authority (Olugbenga Olatunji, Kampala, Uganda, 2 May 2018).

87 Tanzania-Mainland boasts the major pharmaceutical manufacturing capacity in Tanzania, while manufacturing capacity is almost non-existent in Burundi.

88 Six representatives were interviewed, two each from Kenya (Universal Corporation and Cosmos Pharmaceutical Ltd), Uganda (Cipla Quality Chemical and Kampala Pharmaceutical Industry), and Tanzania (ZENUFA Laboratories and Mansoor Daya Pharmaceutical Ltd).

89 *See* Carlson et al. (2005), p. 269; Fromer (2009); Fromer (2016); Furman et al. (2021).

90 *Ibid*.

91 TRIPS Art. 29(1).

92 Patents Act Tanzania-Mainland Sec. 18(6); IPA Zanzibar Sec. 16(4)(a).

93 IPA Kenya Sec. 34(5), as amended by the Statute Law (Miscellaneous Amendments) Act 2008; IPA Uganda Secs. 21(5)(a) and 39(a).

94 IPA Uganda Secs. 21(5)(a) and 39(a).

95 IPA Kenya Sec. 34(5) (as amended).

96 Rwandan Law No. 31/2009 Art. 25.

97 Burundian Law No. 1/13 Art. 20.

98 IPA Kenya Sec. 34(5) (as amended); IPA Uganda Secs. 21(5)(a); Rwandan Law No. 31/2009 Art. 25; Patents Act Tanzania-Mainland Sec. 18(6); Burundian Law No. 1/13 Art. 20; IPA Zanzibar Sec. 16(4)(a).

99 Olatunji *supra* note 10.

100 *Ibid*.

101 On the dilemma of a best mode requirement, *see* generally: Marchese (1992); Markham (2011); Robinson (2012).

102 *See* TRIPS Art. 62(4).

103 *See* generally Soobert (1998).

104 IPA Kenya Sec. 103; Patents Act Tanzania-Mainland Sec. 64; Rwandan Law No. 31/2009 Art. 36.

105 IPA Zanzibar Sec. 10(7)(a); Burundian Law No. 1/13 Art. 48.

106 IPA Uganda Secs. 28(7)–(9), 32(5)–(6).

107 IPA Kenya Sec. 103(3); IPA Uganda Secs. 28(9) and 32(5); Rwandan Law No. 31/2009 Art. 36; Patents Act Tanzania-Mainland Sec. 64(2); Burundian Law No. 1/13 Art. 48; IPA Zanzibar Sec. 10(7)(a).

108 Olatunji *supra* note 10.

109 IPA Uganda Secs. 28(7)–(9), 32(5)–(6).

110 *See* IPA Uganda Sec. 8(3)(f).

111 Olatunji *supra* note 10.

112 *See* generally McKeith (2013); Rai and Jagannathan (2012); Abbott (2007).

113 *Ibid*.

114 *Ibid*.

115 IPA Kenya Sec. 58(2); IPA Uganda Sec. 43(2); IPA Zanzibar Sec. 12(4)(a)(i).

116 Burundian Law No. 1/13 Art. 57(1).

117 *Ibid* Art. 59.

118 *Ibid*.

119 *Ibid* Arts. 60–61.

120 Rwandan Law No. 31/2009 Art. 40; Patents Act Tanzania-Mainland Sec. 38(2).

121 *See* Tanzania Food, Drugs and Cosmetics Act 2003 (Tanzania), Sec. 73(2).

122 Rwandan Law No. 31/2009 Art. 40.

123 Olatunji *supra* note 10.

124 *Ibid*.

125 *Ibid*.

126 For some background on the compulsory-licence-for export regime, *see* Olatunji (2022), p. 402 (particularly footnote 157).

127 On compulsory licensing, *see* generally Lybecker and Fowler (2009); Bird (2009).

128 IPA Kenya Secs. 72–77 and 80; IPA Uganda Secs. 58–63; Rwandan Law No. 31/2009 Arts. 47–55; Patents Act Tanzania-Mainland Secs. 53–60 and 62; Burundian Law No. 1/13 Arts. 78–102; IPA Zanzibar Sec. 14.

129 IPA Uganda Secs. 44(e) and 66(14); IPA Zanzibar Sec. 14(1)(b) and (7); Burundian Law No. 1/13 Arts. 81 and 88.

130 IPA Kenya Sec. 80(1)(a); IPA Uganda Sec. 66(1)(a); Patents Act Tanzania-Mainland Sec. 55(1); Burundian Law No. 1/13 Art. 78(1); IPA Zanzibar Sec. 14(1)(a)(i).

131 Olatunji supra note 10.

132 *Ibid*.

133 IPA Zanzibar Sec. 14(1)(a)(vi).

134 Burundian Law No. 1/13 Art. 86.

135 IPA Kenya Sec. 74(1)(a); IPA Uganda Sec. 60(1)(a); Rwandan Law No. 31/2009 Art. 51; Patents Act Tanzania-Mainland Sec. 56(a).

136 IPA Kenya Sec. 75(2)(e); IPA Uganda Sec. 61(2)(e); Rwandan Law No. 31/2009 Art. 51; Patents Act Tanzania-Mainland Sec. 57(2)(d); Burundian Law No. 1/13 Art. 80; IPA Zanzibar Sec. 14(1)(b).

137 IPA Zanzibar Sec. 73(3).

138 Patents Act Tanzania-Mainland (n 30) Sec. 62(4).

139 Burundian Law No. 1/13 Art. 97.

140 IPA Kenya Secs. 72(1) and 80(1)(b); Patents Act Tanzania-Mainland Secs. 53 and 62; Burundian Law No. 1/13 Arts. 80, 86, 91–95.

141 *See* TRIPS Art. 31(f).

142 TRIPS (as amended) Art. 31^bis^ (3).

143 *See* Appendix to Annex to the TRIPS Agreement, https://www.wto.org/english/docs_e/legal_e/31bis_TRIPS_annex_e.htm.

144 IPA Zanzibar Sec. 14(1)(b) and (7).

145 Burundian Law No. 1/13 Arts. 81 and 88.

146 IPA Uganda Secs. 44(e) and 66(14).

147 Cotter, for instance, identifies three problems: a cumbersome process, behind-the-scenes pressure from developed countries against the use of the regime, and lack of the required infrastructure to manufacture and distribute in developing (and least developed) countries, *see* Cotter (2008); Ho (2011), pp. 214–219; Abbas and Riaz (2008), pp. 9–11; Rimmer 2008.

148 *Ibid*.

149 *See* generally Yu (2023) (forthcoming).

150 *See* for instance Abbas and Riaz *supra* note 147, p. 10.

151 EAC Protocol on TRIPS Flexibilities Sec. 12(2). Interestingly, EAC partner states are currently not parties to any bilateral or free trade agreement which could have required them to enact TRIPS-plus obligations; rather, this embracement of TRIPS-plus obligations is more a function of the fact that LMICs often receive so-called technical assistance from WIPO in drafting their IP legislation.

152 *See* generally Lopert and Gleeson (2013); Roffe and Spennemann (2006).

153 Roffe and Spennemann *supra* note 152.

154 This practice is permissible under TRIPS: *see* TRIPS (n 3) Art. 1(1).

155 IPA Zanzibar (n 17) Sec. 72(5)(d); Burundian Law No. 1/13 (n 20) Art. 378.

156 Interviews: Medical Procurement and Production Division (Olugbenga Olatunji, Kigali, Rwanda, 22 May 2018); Mansoor Daya Pharmaceutical Ltd (n 84); ZENUFA Laboratories (n 84).

157 *See* The Guidelines on the Registration of Pharmaceuticals for Human Use in Uganda (revised July 2006), G 2(1).

158 Interview: Poison and Pharmacy Board (n 81).

159 Burundian Law No. 1/13 (n 20) Art. 62; IPA Zanzibar (n 17) Sec. 13(1)(a).

160 IPA Zanzibar (n 17) Sec. 13(1)(b).

161 *Ibid*.

162 Burundian Law No. 1/13 (n 20) Art. 62.

163 The ongoing negotiations of a free trade agreement between Kenya and the United States could be expected to unleash a whole gamut of TRIPS-plus obligations on the region if successful. Whether this process will be successful, however, remains to be seen, especially in view of an application successfully brought by interested parties before the EAC Court of Justice to stop Kenya proceeding with these negotiations without carrying other EAC partner states along: *see* *Christopher* *Ayieko* *&* *Anor* *v.* *The* *Attorney* *General* *of* *the* *Republic* *of* *Kenya* *&* *Anor* (Reference No. 9 of 2019), judgment delivered on 2 December 2022; *see* *also* “The Proposed Kenya-USA Free Trade Agreement”, https://agoa.info/bilaterals/kenyausa.html.

164 There are currently 283.7 million people living in the region: *see* Overview of EAC. https://www.eac.int/overview-of-eac.

165 EAC PMP *supra* note 4.

166 EAC MRH *supra* note 5.
